# KEAP1/NFE2L2 Mutations of Liquid Biopsy as Prognostic Biomarkers in Patients With Advanced Non-Small Cell Lung Cancer: Results From Two Multicenter, Randomized Clinical Trials

**DOI:** 10.3389/fonc.2021.659200

**Published:** 2021-07-26

**Authors:** Hongyuan Zhu, Daipeng Xie, Yunfang Yu, Lintong Yao, Bin Xu, Luyu Huang, Shaowei Wu, Fasheng Li, Yating Zheng, Xinyi Liu, Wenzhuan Xie, Mengli Huang, Hao Li, Shaopeng Zheng, Dongkun Zhang, Guibin Qiao, Lawrence W. C. Chan, Haiyu Zhou

**Affiliations:** ^1^ Division of Thoracic Surgery, Guangdong Provincial People’s Hospital & Guangdong Academy of Medical Sciences, School of Medicine, South China University of Technology, Southern Medical University, Guangzhou, China; ^2^ Division of Thoracic Surgery, Guangdong Provincial People’s Hospital & Guangdong Academy of Medical Sciences, Guangzhou University of Chinese Medicine, Guangzhou, China; ^3^ Guangdong Provincial Key Laboratory of Malignant Tumor Epigenetics and Gene Regulation, Department of Medical Oncology, Sun Yat-sen Memorial Hospital, Sun Yat-sen University, Guangzhou, China; ^4^ The Medical Department, 3D Medicines Inc., Shanghai, China; ^5^ Division of Thoracic Surgery, Cancer Hospital of Shantou University Medical College, Shantou, China; ^6^ Department of Health Technology and Informatics, The Hong Kong Polytechnic University, Hong Kong, China

**Keywords:** prognostic, KEAP1/NFE2L2, non-small cell lung cancer, immunotherapy, chemotherapy

## Abstract

**Purpose:**

The KEAP1-NFE2L2 (Kelch-like ECH-associated protein 1 (KEAP1)-Nuclear factor (erythroid-derived 2)-like 2 (NFE2L2)) mutations are associated with resistance to chemotherapy or immunotherapy in non-small cell lung cancer (NSCLC). Conversely, it has been reported that NFE2L2 mutations potentiate improved clinical outcome with immunotherapy. However, therapeutic benefits for patients with KEAP1/NFE2L2 mutations remain unclear. The purpose of this study was to investigate the association between KEAP1/NFE2L2 and NSCLC prognosis, and to explore whether immunotherapy can improve prognosis in populations with KEAP1/NFE2L2 mutations.

**Experimental Design:**

The impact of KEAP1/NFE2L2 mutations on survival outcomes in NSCLC patients received immunotherapy and chemotherapy was verified in the randomized phase II/III POPLAR/OAK trials (blood-based sequencing, bNGS cohort, POPLAR (n = 211) and OAK (n = 642)). The Cancer Genome Atlas (TCGA) NSCLC cohort (n=998) and an in-house Chinese NSCLC cohort (n=733) was used For the analysis of immune-related markers.

**Results:**

Compared with KEAP1/NFE2L2 wild-type, patients with KEAP1/NFE2L2 mutations were significantly associated with poorer overall survival (OS, HR = 1.97, 95% CI 1.48–2.63, *P* < 0.001) on atezolizumab and docetaxel (HR = 1.66, 95% CI 1.28–2.16, *P* < 0.001). In KEAP1/NFE2L2 mutant group, there was no significant difference in median OS between atezolizumab and docetaxel (HR 0.74, 95% CI 0.53–1.03, *P* = 0.07). NFE2L2/KEAP1 mutations were significantly associated with higher TMB values and PD-L1 expression in the OAK/POPLAR and in-house Chinese NSCLC cohorts. GSEA revealed that KEAP1/NFE2L2mutant subgroup was associated with deficient infiltration of CD4+ T cells, NK T cells and natural Treg cells, and lower expression of DNA damage response genes in TCGA NSCLC cohort.

**Conclusions:**

Our study revealed that patients with KEAP1/NFE2L2 mutations have a worse prognosis than wild-type patients, both on immunotherapy and chemotherapy. In addition, in patients with KEAP1/NFE2L2 mutations, immunotherapy did not significantly improve prognosis compared to chemotherapy.

## Introduction

The Kelch-like ECH-associated protein 1 (KEAP1)/nuclear factor erythroid-2-related factor 2 (NRF2, also known as NFE2L2) pathway plays a critical role in the oxidative stress response (1). Mutations in this pathway are common in non-small cell lung cancer (NSCLC) and have been associated with enhanced tumor growth and aggressiveness (2). With the development of evidence on the role of KEAP1/NFE2L2 pathway on chemotherapy resistance in pre-clinical NSCLC models (3), there have been increasing efforts to evaluate the impact of KEAP1/NFE2L2 mutations on the prognosis of survival in NSCLC patients.

The effect of KEAP1/NFE2L2 mutations on chemotherapy efficacy has been examined in several studies. In a retrospective cohort study of over 1400 NSCLC patients from the Regina Elena National Cancer Institute, Memorial Sloan Kettering Cancer Center and The Cancer Genome Atlas (TCGA) network, it is found that KEAP1/NFE2L2 mutations represent a mechanism of intrinsic resistance to chemotherapy (4). A retrospective cohort study of 103 NSCLC patients, found a poor survival in patients with KEAP1/NFE2L2 mutations. Consistent with the above study results, other study had also demonstrated that KEAP1/NFE2L2 mutation reduces response rate and survival in NSCLC patients receiving chemotherapy (5).

In terms of immunotherapy, a previous study of 550 NSCLC patients from Memorial Sloan Kettering, has shown that patients with KEAP1/NFE2L2 co-mutation in KRAS have significantly shorter survival (6). A letter to the editor present a cohort of 69 patients from the Memorial Sloan Kettering Sequencing with KEAP1/NFE2L2 mutations who were treated with immunotherapy and demonstrated inferior survival compared with patients with KEAP1/NFE2L2 wild-type tumors (7).Similar results were also described in KEAP1 mutations of a prospective study with 66 NSCLC patients, which are unresponsive to immunotherapy (8). In the immunotherapy group of MYSTIC trial, patients with KEAP1/NFE2L2 mutations had poor overall survival compared with KEAP1/NFE2L2 wild-type patients (9). Congruent with prior studies, KEAP1 mutations were associated with significantly shorter overall survival (OS) in patients receiving immunotherapy (10, 11). However, the impact of KEAP1/NFE2L2 mutation on the efficacy of immunotherapy in advanced NSCLC remains a controversial topic. There has been conflicting evidence on the role of KEAP1/NFE2L2 mutations in ICI response with several series reporting better response to ICI. For example, a small (N=34) retrospective analysis found that KEAP1 mutations were more frequent in pembrolizumab-treated NSCLC patients who had sustained clinical benefits (12). In addition, an exploratory analysis of the KEYNOTE-042 trial presented at the AACR 2020 meeting suggested a possible role for ICI therapy in KEAP1-mutant NSCLC (13). Based on the above study, the clinical significance of KEAP1/NFE2L2 mutations in immunotherapy remains elusive and somewhat contradictory.

Since many previous studies have shown that KEAP1/NFE2L2 mutation is associated with poor prognosis of NSCLC, it is of great clinical significance to determine which treatment method can improve the prognosis of NFE2L2/KEAP1 mutant population. A previous study of NSCLC patients with KEAP1/NFE2L2 mutations from Memorial Sloan Kettering Cancer Center database, has shown that KEAP1/NFE2L2 mutations were associated with inferior overall survival but improved survival in the KEAP1-NFE2L2 mutant tumors treated with immunotherapy compared with other treatments (14). However, no prospective study datas have been retrospectively used to compare the outcomes of patients with KEAP1/NFE2L2 mutations who receive immunotherapy versus chemotherapy.

Therefore, in order to evaluate the prognostic effect of KEAP1/NFE2L2 mutations on immunotherapy and chemotherapy using data from two independent cohorts (the prospective randomized phase II/III POPLAR/OAK trials with both immunotherapy and chemotherapy groups) were used to analyze the prognostic effect of KEAP1/NFE2L2 mutations on atezolizumab and docetaxel, and the correlation with immunogenic markers. In addition, TCGA data and Chinese real world data were used to analyze prognostic mechanisms. Our findings suggest that the KEAP1/NFE2L2 mutations may be a poor prognostic biomarker for NSCLC, and compared with docetaxel, atezolizumab did not prolong OS in patients with KEAP1/NFE2L2 mutations.

## Materials and Methods

### Clinical Cohorts and Study Design

We searched literatures and found two NSCLC cohorts treated with anti-PD-L1 and docetaxel, which were POPLAR study (phase II trial, NCT01903993) and OAK study (phase III trial, NCT02008227), with 211 and 642 patients had blood-based next-generation sequencing data (Foundation One panel), respectively. We retrieved the targeted-sequencing and clinical data of these patients (n=853) from previously published article (PMID: 30082870) (15). Survival analysis were conducted to explore the prognostic value of mutations in these two cohorts. To further explore the transcriptome mechanism, the mRNA expression data from Cancer Genome Atlas (TCGA) NSCLC cohort (n=998) were also included for Gene set enrichment analysis (GSEA, see below). We obtained the WES and mRNA expression data from the GDC data portal (https://portal.gdc.cancer.gov/projects). To clarify the immune-related characteristics, we compare the PD-L1 and TMB levels of mutational subgroups in the OAK/POPLAR cohorts and an in-house Chinese NSCLC cohort (n=733).

### KEAP1/NFE2L2 Mutations

Nonsynonymous mutations including TRUNC (frameshift del, frameshift ins, nonsense, nonstop, splice region, and splice site), INFRAME (inframe del and inframe ins), and MISSENSE mutations of KEAP1/NFE2L2 were defined as KEAP1/NFE2L2 mutations in this study.

### Gene Set Enrichment Analysis (GSEA)

GSEA was performed in the TCGA NSCLC cohort using gene sets from Molecular Signature Database (MSigDB) v.7.0 to investigate the biological features of KEAP1/NFE2L2 mutant NSCLC (16). GSEA was conducted by clusterProfilter R package (17). The *P*-value estimates the statistical significance of the normalized enrichment score (NES). A gene set with *P* < 0.05 was determined to be significantly enriched in genes.

### Statistical Analysis

Continuous variables were compared by the Mann Whitney U test and categorical variables were compared using chi-squared or Fisher’s exact tests. The Kaplan–Meier method was used to delineate the OS curve, and the log-rank method was used to assess the significance. The hazard ratio (HR) was determined through the univariable and multivariable Cox regression. Variables with a *P*-value below 0.10 in the univariable regression were included in the multivariable analyses.

**Figure 1:**
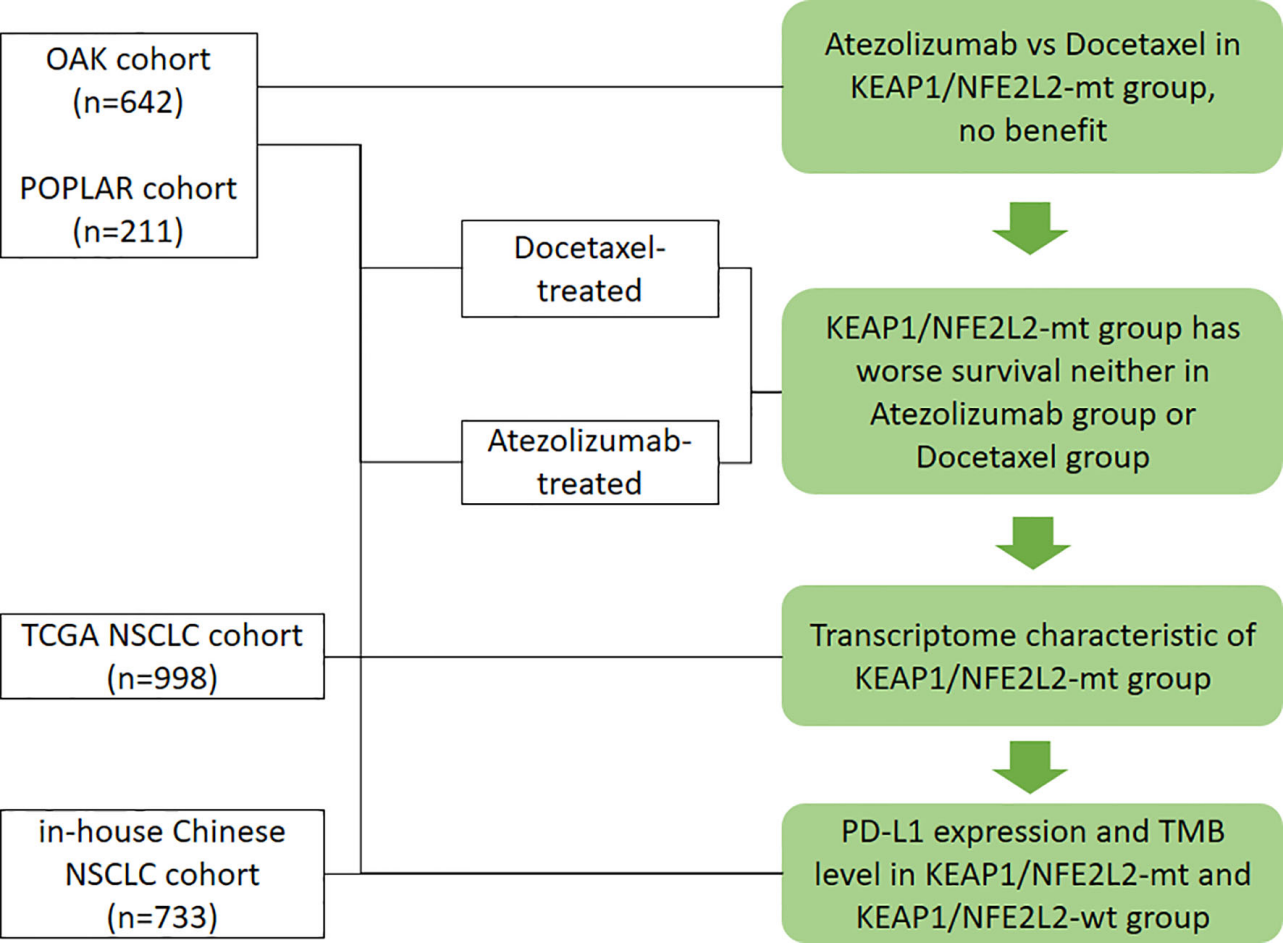
Workflow of the study. Flow diagram illustrating the cohorts considered and the main purposes of the analytical process.

All analyses and graphs were performed using R 3.6.3 (R Foundation for Statistical Computing, Vienna, Austria). If not specified, tests were two-tailed, and a *P*-value of <0.05 was considered statistically significant. The workflow of the study is illustrated in **Figure 1**.

## Results

### Characteristics of the Patients and Mutational Patterns

A pooled analysis of two independent cohorts of 853 advanced NSCLC patients showed that 171 (20.0%) harbored KEAP1/NFE2L2 mutations, comprising 211 patients from the POPLAR trial (49 [23.2%] with KEAP1/NFE2L2 mutations) and 642 from the OAK trial (122 [19.0%] with KEAP1/NFE2L2 mutations). KEAP1/NFE2L2 had higher mutation frequency in lung squamous cell carcinoma (OAK/POPLAR, *P* = 0.05; OAK, *P* = 0.044; POPLAR, *P* = 0.069). A high proportion of smokers were found in patients with KEAP1/NEF2L2 mutations (OAK/POPLAR, *P* < 0.001; OAK, *P* < 0.001; POPLAR, *P* = 0.009). The characteristics of patients with KEAP1/NEF2L2 mutations versus wild-type in the overall cohort are shown in **Table 1**. The genomic mutational landscape of 853 patients showed that KEAP1 or NEF2L2 mutations prevailed in 118 (14%) or 58 (7%) (**Supplementary Figure 1**), respectively.

**Table 1:** Clinical characteristics of patients with NFE2L2/KEAP1 mutations treated with atezolizumab or docetaxel in the OAK/POPLAR cohorts.

	OAK (n=642)		*P* value	POPLAR (n=211)		*P* value	Total (n=853)		*P* value
	KEAP1/NFE2L2(+) n=122	KEAP1/NFE2L2(-) n=520		KEAP1/NFE2L2(+) n=49	KEAP1/NFE2L2(-) n=162		KEAP1/NFE2L2(+) n=171	KEAP1/NFE2L2(-) n=682	
**Median age (interquartile rang), years**	63 (57-69)	64 (57-70)	0.847	59 (53-67)	62 (52-69)	0.132	62 (57-68)	64 (57-70)	0.289
**Gender, n (%)**									
** Female**	26 (21.3)	219 (42.1)	<0.001**	17 (34.7)	64 (39.5)	0.544	43(25.1)	283 (41.5)	0.001**
** Male**	96 (78.7)	301 (57.9)		32 (65.3)	98 (60.5)		128(74.9)	399 (58.5)	
**Histology subtype, n (%)**									
** Squamous**	43 (35.2)	136 (26.2)	0.044*	23 (46.9)	53 (32.7)	0.069	66(38.6)	189 (27.7)	0.05
** Non-Squamous**	79 (64.8)	384 (73.8)		26 (53.1)	109 (67.3)		105(61.4)	493 (72.3)	
**Smoking history, n (%)**									
** Current**	27 (22.1)	67 (12.9)	<0.001**	13 (26.5)	31 (19.1)	0.009*	40 (23.4)	98 (14.4)	<0.001**
** Previous**	90 (73.8)	353 (67.9)		35 (71.4)	97 (59.9)		125 (73.1)	450 (66.0)	
** Nevers**	5 (4.1)	100 (19.2)		1 (2.0)	34 (21.0)		6(3.5)	134(19.6)	
**Race, n (%)**									
** White**	88 (72.1)	361 (69.4)	0.199	46 (93.9)	121 (74.7)	0.013*	134 (78.4)	482 (70.7)	0.036*
** Asian**	20 (16.4)	118 (22.7)		1 (2.0)	25 (15.4)		21 (12.3)	143 (21.0)	
** Other**	14 (11.5)	41 (7.9)		2 (4.1)	16 (9.9)		16 (9.3)	57 (8.3)	
**Median BLSLD (interquartile rang), mm**	80 (51-118)	67 (42-99)	0.041*	79 (51-111)	72 (51-121)	0.914	77 (51-118)	72 (44-103)	0.06
**Median bTMB (interquartile range), mutations/Mb**	14.5 (9-21)	6 (3-13)	<0.001**	16 (9-22)	6 (3-14.75)	<0.001**	15 (9-22)	6 (3-13)	<0.001*
**PD-L1 expression, n (%)**									
** PD-L1＜50%**	100	428	0.4967						
** PD-L1≥50%**	22	89							
** Unknown**	0	3							
**KRAS status (%)**									
** Mutation**	12 (9.8)	49 (9.4)	0.889	6 (10.2)	6 (3.7)	0.056	18(10.5)	55(8.1)	0.278
** Wild type**	110 (90.2)	471 (90.6)		43 (89.8)	156 (96.3)		153(89.5)	627(91.9)	
**TP53 status (%)**									
** Mutation**	79 (64.8)	232 (44.6)	<0.001**	33 (67.3)	76 (46.9)	0.012*	112(65.5)	308(45.2)	<0.001**
** Wild type**	43 (35.2)	288 (55.4)		16 (32.7)	86 (53.1)		59((34.5)	374(54.8)	
**STK11**									
** Mutation**	21 (17.2)	21 (4.0)	<0.001**	10 (20.4)	12 (7.4)	0.009*	31(18.1)	33(4.8)	<0.001**
** Wild type**	101 (82.8)	499 (96.0)		39 (79.6)	150 (92.6)		140(81.9)	649(95.2)	
**EGFR status (%)**									
** Mutation**	4 (3.3)	51 (9.8)	0.02*	3 (6.1)	20 (12.3)	0.221	7(4.1)	71(10.4)	0.01*
** Wild type**	118 (96.7)	469 (90.2)		46 (93.9)	142 (87.7)		164(95.9)	611(89.6)	
**ALK status (%)**									
** Mutation**	9 (7.4)	11 (2.1)	0.007	4 (8.2)	10 (6.2)	0.871	13(7.6)	21(3.1)	0.007*
** Wild type**	113 (92.6)	509 (97.9)		45 (91.8)	152 (93.8)		158(92.4)	661(96.9)	

BLSLD, baseline sum of the longest diameters; bTMB, blood tumor mutational burden; PD-L1, programmed cell death 1 ligand 1; *P ＜ 0.05; **P ＜ 0.005.

### Immunotherapy and Chemotherapy Are Not Effective in Patients With NFE2L2/KEAP1 Mutations

**Figure 2:**
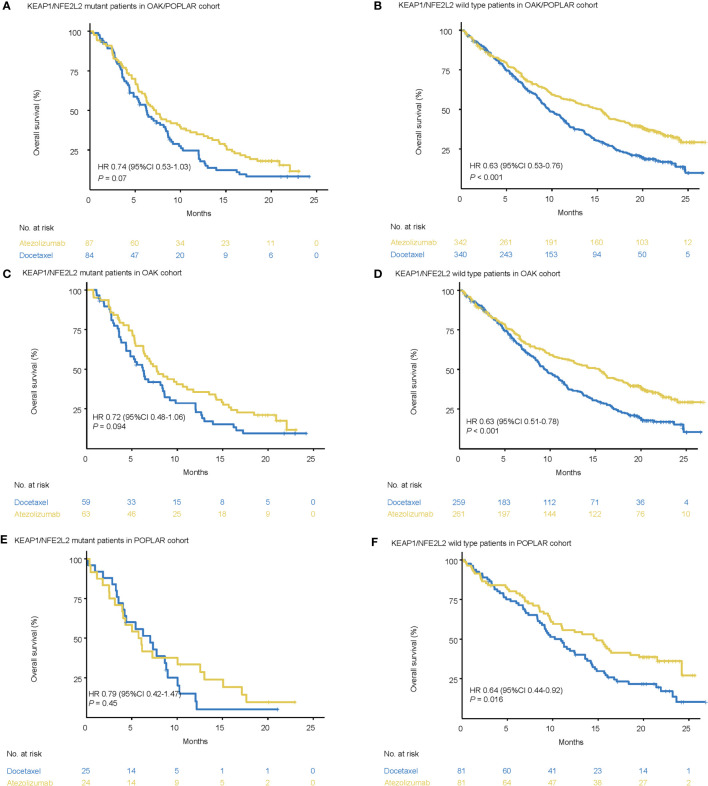
Survival analyses in the OAK/POPLAR cohorts. **(A)** Kaplan–Meier curves of overall survival (OS) in the patients with KEAP1/NFE2L2 mutations who received atezolizumab and docetaxel in the OAK/POPLAR cohorts. **(B)** Kaplan–Meier curves of OS in the patients without KEAP1/NFE2L2 mutations who received atezolizumab and docetaxel in the OAK/POPLAR cohorts. **(C)** Kaplan–Meier curves of OS in the patients with KEAP1/NFE2L2 mutations who received atezolizumab and docetaxel in the OAK cohort. **(D)** Kaplan–Meier curves of OS in the patients without KEAP1/NFE2L2 mutations who received atezolizumab and docetaxel in the OAK cohort. **(E)** Kaplan–Meier curves of OS in the patients with KEAP1/NFE2L2 mutations who received atezolizumab and docetaxel in the POPLAR cohort. **(F)** Kaplan–Meier curves of the OS in the patients without KEAP1/NFE2L2 mutations who received atezolizumab and docetaxel in the POPLAR cohort.

In the KEAP1/NFE2L2-mutant cohort, the median OS was not significantly different between docetaxel and atezolizumab. (OAK/POPLAR, HR = 0.74, 95% CI 0.53–1.03, *P* = 0.07; OAK, HR = 0.72, 95% CI 0.48–1.06, *P* = 0.094; POPLAR, HR = 0.79, 95% CI 0.42–1.47, *P* = 0.45) (**Figures 2A, C, E**).

However, in the KEAP1/NFE2L2 wild-type cohort, OS for patients treated with atezolizumab was longer than patients treated with docetaxel (OAK/POPLAR, HR = 0.63, 95% CI 0.53–0.76, *P* < 0.001; OAK, HR = 0.63, 95% CI 0.51–0.78, *P* < 0.001, POPLAR, HR = 0.64, 95% CI 0.44–0.92, *P* = 0.016) (**Figures 2B, D, F**).

When EGFR and ALK mutations were excluded, survival analysis in KEAP1/NFE2L2 mutant cohorts indicated no significant difference in OS between patients treated with atezolizumab or docetaxel (OAK/POPLAR, HR = 0.74, 95% CI 0.52–1.05, *P* = 0.095; OAK, HR = 0.73, 95% CI 0.48–1.1, *P* = 0.13; POPLAR, HR = 0.73, 95% CI 0.35–1.4, *P* = 0.31) (**Supplementary Figures 2A, C, E**). However, analysis in the KEAP1/NFE2L2 wild-type cohort showed that the OS for patients treated with atezolizumab was longer than that for patients treated with docetaxel (OAK/POPLAR, HR = 0.6, 95% CI 0.5–0.73, *P* < 0.001; OAK, HR = 0.6, 95% CI 0.48–0.75, *P* < 0.001; POPLAR, HR = 0.62, 95% CI 0.41–0.94, *P* = 0.025) (**Supplementary Figures 2B, D, F**).

### KEAP1/NFE2L2 Mutations Are Associated With Worse Immunotherapy Survival in NSCLC

In the OAK/POPLAR-atezolizumab cohort, KEAP1/NEF2L2 mutations (55.7%) were associated with a higher disease control rate (DCR) (OAK/POPLAR, *P* = 0.0074) than KEAP1/NEF2L2 wild-type (38.5%) (**Supplementary Figure 3A**).

**Figure 3:**
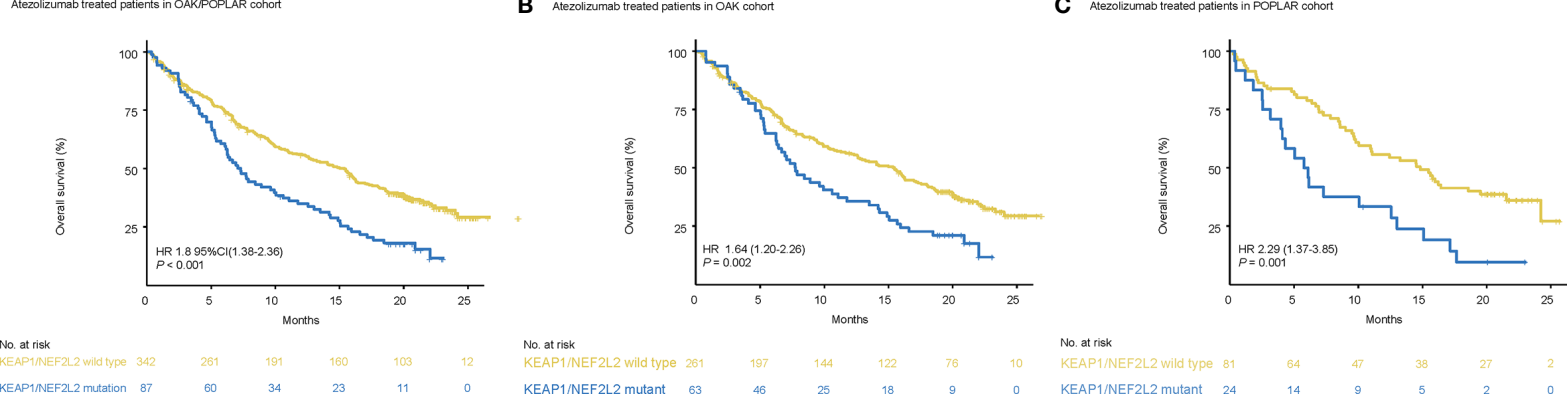
Survival analyses in the OAK/POPLAR-atezolizumab cohorts. **(A)** Kaplan–Meier curves of overall survival (OS) in the atezolizumab treated patients with or without KEAP1/NFE2L2 mutations in the OAK/POPLAR cohorts. **(B)** Kaplan–Meier curves of OS in the atezolizumab treated patients with or without KEAP1/NFE2L2 mutations in the OAK cohort. **(C)** Kaplan–Meier curves of OS in the atezolizumab treated patients with or without KEAP1/NFE2L2 mutations in the POPLAR cohort.

Patients with KEAP1/NEF2L2 mutations had a significantly shorter median OS than KEAP1/NFE2L2 wild-type patients (OAK/POPLAR, HR = 1.97, 95% CI 1.48–2.63, *P* < 0.001) (**Figure 3A**). In addition, the survival analysis in the OAK-atezolizumab cohort and POPLAR-atezolizumab cohort showed that patients with KEAP1/NFE2L2 mutations exhibited shorter OS than KEAP1/NFE2L2 wild-type (OAK, HR = 1.8, 95% CI 1.28–2.52, P<0.001; POPLAR, HR = 2.44, 95% CI 1.42–4.2, P<0.001) (**Figures 3B, C**). When EGFR and ALK mutations were excluded, survival analysis in the OAK/POPLAR-atezolizumab with the EGFR/ALK wild-type cohort, OAK atezolizumab with EGFR/ALK wild-type cohort, and POPLAR-atezolizumab with EGFR/ALK wild-type cohort showed that patients with KEAP1/NFE2L2 mutations still exhibited shorter OS than those with KEAP1/NFE2L2 wild-type (OAK/POPLAR, HR = 1.97, 95% CI 1.48–2.63, P < 0.001; OAK, HR = 1.8, 95% CI 1.28–2.52, P<0.001; POPLAR, HR = 2.44, 95% CI 1.42–4.2, P<0.001) (**Supplementary Figure 4**).

In univariate analyses, KEAP1/NFE2L2 mutations, race, smoking status, histological and pathological types (HIST), bTMB, baseline serum lactate dehydrogenase (SLD), and PD-L1 status were significantly correlated with OS (**Table 2**). In multivariate analyses, KEAP1/NFE2L2 mutations, HIST, baseline SLD, and PD-L1 status were significantly correlated with OS (**Table 2**).

**Table 2:** Hazard ratio (HR) of clinical and genomic variables on overall survival *via* univariate and multivariate analysis in patients treated with Atezolizumab.

Variable	POPLAR cohort – Atezolizumab (n=105)			
	Univariate		Multivariate	
	HR (95% Cl)	P	HR (95% Cl)	P
SEX	1.17 (0.7-1.93)	0.55	
Age (>60 *vs* ≤60)	1.1 (0.69-1.76)	0.684	
Race (white *vs* non- white)	1.79 (1-3.22)	0.051	
Smoking (smoker *vs* non-smoker)	2.14 (0.98-4.67)	0.057	
HIST (non-squamous *vs* squamous)	0.8 (0.49-1.3)	0.361	
TMB (>median *vs* <median)	1.3 (0.84-2.1)	0.229	0.95 (0.56-1.6)	0.832
baseline SLD (>median *vs* <median)	1.7 (1.1-2.7)	0.026	1.82 (1.1-2.9)	0.013
SRP status (mut *vs* wt)	2.29 (1.37-3.85)	0.002	2.52 (1.42-4.5)	0.002
**Variable**	**OAK cohort – Atezolizumab (n=324)**			
	**Uni-variate**		**Multi-variate**	
	**HR (95% Cl)**	**P**	**HR (95% Cl)**	**P**
SEX	1.27 (0.96-1.69)	0.092		
Age (>60 *vs* ≤60)	0.94 (0.71-1.24)	0.662	
Race (white *vs* non- white)	1.25 (0.92-1.69)	0.149	
Smoking (smoker *vs* non-smoker)	1.27 (0.88-1.82)	0.197	
HIST (non-squamous *vs* squamous)	0.68 (0.51-0.91)	0.009	
TMB (>median *vs* <median)	1.48 (1.1-1.9)	0.004	1.26 (0.93-1.70)	0.135
baseline SLD (>median *vs* <median)	1.8 (1.4-2.4)	<0.001	1.63 (1.23-2.17)	<0.001
SRP status (mut *vs* wt)	1.64 (1.2-2.26)	0.002	1.32 (0.94-1.85)	0.113
**Variable**	**POPLAR/OAK cohort – Atezolizumab (n=429)**			
	**Uni-variate**		**Multi-variate**	
	**HR (95% Cl)**	**P**	**HR (95% Cl)**	**P**
SEX	1.25 (0.98-1.6)	0.0784		
Age (>60 *vs* ≤60)	0.98 (0.77-1.24)	0.8542		
Race (white *vs* non-white)	1.36 (1.04-1.78)	0.0245	1.23 (0.93-1.63)	0.152
Smoking (smoker *vs* non-smoker)	1.43 (1.03-1.98)	0.0342	1.08 (0.76-1.55)	0.661
HIST (squamous *vs* non-squamous)	1.41 (1.1-1.81)	0.0067	1.29 (1-1.67)	0.051
bTMB (>median *vs* <median)	1.44 (1.14-1.82)	0.0023	1.05 (0.81-1.36)	0.706
baseline SLD (>median *vs* <median)	1.78 (1.41-2.26)	<0.001	1.68 (1.31-2.14)	<0.001
SRP status (mut *vs* wt)	1.8 (1.38-2.36)	<0.001	1.56 (1.16-2.08)	0.003

Subgroup analysis based on PD-L1 expression level showed OS benefit in the KEAP1/NFE2L2 wild-type group was significant in patients with PD-L1 < 50% (PD-L1 < 50%, HR = 1.77, 95% CI 1.23–2.55, *P* = 0.01; PD-L1 ≥ 50%, HR = 1.86, 95% CI 0.74–4.66, *P* = 0.13) (**Supplementary Figure 5**).

### KEAP1/NFE2L2 Mutations Associated With Worse Chemotherapy Survival in NSCLC

In the OAK/POPLAR-docetaxel cohort, KEAP1/NEF2L2 mutations (45.8%) were associated with lower DCR (OAK/POPLAR, *P* = 0.0017) than KEAP1/NEF2L2 wild-type (66.7%) (**Supplementary Figure 3B**).

**Figure 4:**
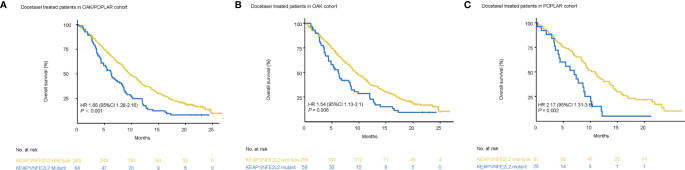
Survival analyses in the OAK/POPLAR-docetaxel cohorts. **(A)** Kaplan–Meier curves of overall survival (OS) in the docetaxel treated patients with or without KEAP1/NFE2L2 mutations in the OAK/POPLAR cohort. **(B)** Kaplan–Meier curves of OS in the docetaxel treated patients with or without KEAP1/NFE2L2 mutations in the OAK cohort. **(C)** Kaplan–Meier curves of OS in the docetaxel treated patients with or without KEAP1/NFE2L2 mutations in the POPLAR cohort.

Survival analysis showed that worse OS was observed in patients with KEAP1/NFE2L2 mutations than in the KEAP1/NFE2L2 wild-type group (OAK/POPLAR, HR = 1.66, 95% CI 1.28–2.16, *P*<0.001) (**Figure 4A**). In the OAK and POPLAR-docetaxel cohort, patients with KEAP1/NFE2L2 mutations had a significantly worse OS than those in the KEAP1/NFE2L2 wild-type group (OAK, HR = 1.54, 95% CI 1.13–2.1, P = 0.006; POPLAR, HR = 2.17, 95% CI 1.31–3.6, P = 0.002) (**Figures 4B, C**).

When EGFR and ALK mutations were excluded, patients with KEAP1/NFE2L2 mutations associated with OAK/POPLAR-docetaxel exhibited shorter OS than the KEAP1/NFE2L2 wild-type group (OAK/POPLAR, HR = 1.68, 95% CI 1.27–2.22, P < 0.001; OAK, HR = 1.56, 95% CI 1.13–2.15, P = 0.0068; POPLAR, HR = 2.34,95% CI 1.31–4.19 P = 0.0033) (**Supplementary Figure 6**).

In univariate analyses, KEAP1/NFE2L2 mutations, race, smoking status, histological and pathological types (HIST), bTMB, and baseline serum lactate dehydrogenase (SLD) were significantly correlated with OS (**Table 3**). In multivariate analyses, KEAP1/NFE2L2 mutations, bTMB, baseline SLD, and PD-L1 status were significantly correlated with OS (**Table 3**).

**Table 3:** Hazard ratio (HR) of clinical and genomic variables on overall survival *via* univariate and multivariate analysis in patients treated with Docetaxel.

Variable	POPLAR cohort – Docetaxel (n=106)			
	Uni-variate		Multi-variate	
	HR (95% Cl)	P	HR (95% Cl)	P
SEX (male *vs* female)	1.53 (0.99-2.36)	0.0531		
Age (>60 *vs* ≤60)	1.04 (0.68-1.59)	0.8591		
Race (white *vs* non- white)	1.55 (0.85-2.8)	0.1497		
Smoking (smoker *vs* non-smoker)	1.67 (0.92-3.02)	0.0903		
HIST (non-squamous *vs* squamous)	1.19 (0.77-1.84)	0.4217		
bTMB (>median *vs* <median)	1.92 (1.25-2.96)	0.0029	1.65 (1.03-2.64)	0.037
baseline SLD (>median *vs* <median)	1.4 (0.92-2.14)	0.1153		
SRP status (mut *vs* wt)	2.17 (1.31-3.6)	0.0026	1.69 (0.98-2.93)	0.059
**Variable**	**OAK cohort – Docetaxel (n=318)**			
	**Uni-variate**		**Multi-variate**	
	**HR (95% Cl)**	**P**	**HR (95% Cl)**	**P**
SEX (male *vs* female)	1.15 (0.89-1.48)	0.2953		
Age (>60 *vs* ≤60)	1.26 (0.97-1.64)	0.0845		
Race (white *vs* non- white)	1.26 (0.96-1.66)	0.0979		
Smoking (smoker *vs* non-smoker)	1.3 (0.9-1.88)	0.158		
HIST (non-squamous *vs* squamous)	1.43 (1.09-1.86)	0.0095	1.29 (0.98-1.69)	0.069
bTMB (>median *vs* <median)	1.57 (1.22-2.01)	<0.001	1.31 (1-1.73)	0.054
baseline SLD (>median *vs* <median)	1.55 (1.21-2)	<0.001	1.39 (1.07-1.81)	0.013
SRP status (mut *vs* wt)	1.54 (1.13-2.1)	0.0066	1.27 (0.91-1.77)	0.166
**Variable**	**POPLAR/OAK cohort – Docetaxel (n=424)**			
	**Uni-variate**		**Multi-variate**	
	**HR (95% Cl)**	**P**	**HR (95% Cl)**	**P**
SEX	1.23 (0.99-1.54)	0.0634		
Age (>60 *vs* ≤60)	1.21 (0.97-1.51)	0.0992		
Race (white *vs* non-white)	1.32 (1.03-1.7)	0.0268	1.15 (0.13-1.08)	0.279
Smoking (smoker *vs* non-smoker)	1.41 (1.03-1.93)	0.0305	1.06 (0.18-0.34)	0.736
HIST (squamous *vs* non-squamous)	1.36 (1.08-1.71)	0.008	1.25 (0.12-1.83)	0.067
bTMB (>median *vs* <median)	1.64 (1.32-2.03)	<0.001	1.34 (0.12-2.36)	0.018
baseline SLD (>median *vs* <median)	1.5 (1.21-1.87)	0.0002	1.33 (0.11-2.52)	0.012
SRP status (mut *vs* wt)	1.66 (1.28-2.16)	0.0002	1.33 (0.15-1.99)	0.047

Subgroup analysis based on PD-L1 expression level indicated a significantly negative impact on KEAP1/NFE2L2 mutations on OS in the PD-L1<50% subgroup (PD-L1<50%, *P* = 0.0062; PD-L1 ≥ 50%, *P* = 0.53) (**Supplementary Figure 7**).

### NFE2L2/KEAP1 Mutations Were Associated With Higher TMB Values and PD-L1 Expression in the OAK/POPLAR and In-House Chinese NSCLC Cohorts

In the OAK/POPLAR cohorts, patients with KEAP1/NFE2L2 mutations had a greater TMB than those with KEAP1/NFE2L2 wild-type (OAK/POPLAR, P<0.0001; OAK, P<0.0; POPLAR, P < 0.0001) (**Supplementary Figures 8A-C**). In the in-house Chinese NSCLC cohort, NFE2L2/KEAP1 mutations significantly correlate with higher TMB value (P<0.0001 (**Supplementary Figure 8D**). KEAP1/NFE2L2 mutations was associated with numerically better PD-L1 expression in the OAK cohort (P= 0.69) (**Supplementary Figure 9A**). In the in-house Chinese NSCLC cohort, KEAP1/NFE2L2 mutations showed significant correlation with higher PD-L1 expression level (P=0.03) (**Supplementary Figure 9B**).

### KEAP1/NFE2L2 Mutations Are Associated With a Low Immune Infiltration and Low Expression of DNA Damage Response (DDR) Genes in TCGA NSCLC Cohort

**Figure 5:**
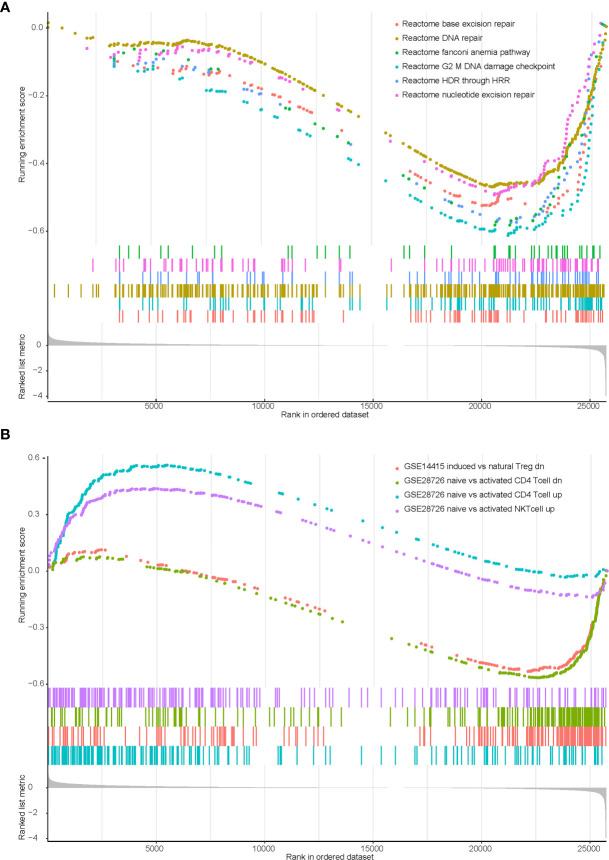
Enrichment plot showing biological signatures based on gene set enrichment analysis (GSEA) of KEAP1/NFE2L2 mutant NSCLC. **(A)**: Significant down-regulation in multiple DDR-related pathways including base excision repair, DNA repair, fanconi anemia pathway, G2 M DNA damage checkpoint, HDR through HRR and nucleotide excision repair. **(B)**: Significant down-regulation in CD4 T cell (the green and blue curves) and NK T cell (the purple curve), significant up-regulation in T regulation cell (the pink curve).

We previously identified that KEAP1/NFE2L2 mutations were irrelevant to PD-L1 expression but associated with higher TMB. Therefore, we speculated that other potential mechanisms may contribute to worse clinical benefit in patients with mutant KEAP1/NFE2L2. We conducted gene set enrichment analysis (GSEA) using transcriptome data of TCGA NSCLC cohort. Significant enrichment scores were observed in DDR related and T cell related pathways (**Supplementary Table 1**). Our results revealed that DNA damage repair related functions were downregulated in KEAP1/NFE2L2 mutant NSCLC. This mutant subgroup was also with deficient infiltration of CD4+ T cells, NK T cells and natural Treg cells (**Figure 5**).

## Discussion

In this report, we analyzed the prognostic association between KEAP1/NFE2L2 mutations and immunotherapy and chemotherapy in 853 patients from the OAK and POPLAR cohort. As previously reported (10), the analysis revealed NFE2L2/KEAP1 were frequently mutated in NSCLC. Previous studies have demonstrated that KEAP1/NFE2L2 mutation reduces response rate and survival in NSCLC patients receiving chemotherapy or immunotherapy. However, the prognosis of KEAP1/NFE2L2 mutations in immunotherapy remains contradictory (12, 13).

In this study, we found that KEAP1/NFE2L2 mutations were associated with poorer survival in NSCLC patients receiving atezolizumab or docetaxel, while there was no significant difference in survival between atezolizumab and docetaxel. These results revealed that patients with KEAP1/NFE2L2 mutations may not benefit from the treatment that is available at present compared with wild-type patients.

Our research showed that KEAP1/NFE2L2 mutations in NSCLC patients were associated with poor OS in chemotherapy. These results corroborate the proposals of Goeman et al., who suggested that KEAP1/NFE2L2 mutations played an important role in NSCLC progression and chemotherapy resistance (4). Jeong et al. revealed that the KEAP1 deletion conferred chemoresistance in murine lung cancer cells and mutations in KEAP1/NFE2L2/CUL3 were associated with worse outcomes after first-line chemotherapy (3). Chemotherapeutic drugs can kill cancer cells *via* the generation of reactive oxygen species (ROS) and subsequent DNA damage. KEAP1/NFE2L2 mutations lead to the constitutive activation of NF2E2L2, promoting cellular resistance to oxidative stress, proliferation, and metabolic reprogramming (18), which may be the possible mechanisms of chemotherapy resistance.

TMB and PD-L1 are widely recognized as prognostic biomarkers for immunotherapy (18, 19). Xu et al. reported that all patients who harbored KEAP1/NFE2L2 mutations had higher TMB values and PD-L1 expression in pan-cancer (20). Our study showed consistent results, where the significantly higher TMB values and PD-L1 expression were associated with KEAP1/NFE2L2 mutation compared with KEAP1/NFE2L2 wild-type in NSCLC. Perhaps the most clinically relevant finding is that the OS was shorter in patients treated with atezolizumab with mutated KEAP1/NFE2L2 than in those with wild-type KEAP1/NFE2L2 independent of TMB and PD-L1. This proves that KEAP1/NFE2L2 mutations affect immunotherapy by another mechanism.

In recent years, studies on the tumor immune microenvironment have been at the forefront of cancer research (19). The effect of the tumor immune microenvironment on immunotherapy has been well reported in many cancer types (20, 21). Cai et al. indicated that defective tumor angiogenesis and lack of adequate immune-cell infiltration were the immunological properties of patients with mutated KEAP1/NFE2L2 (22). Our study showed consistent results that mutated KEAP1/NFE2L2 patients lack infiltration of CD4+ T cells, NK T cells and natural Treg cells, which could be the reason that patients with KEAP1/NFE2L2 mutations have a poor response to immunotherapy. ROS is a group of short-lived, highly reactive, oxygen-containing molecules that can induce DNA damage (23). However, KEAP1/NFE2L2 mutation leads to structural activation of NFE2L2, which promotes cellular resistance to oxidative stress and can balance ROS levels, thus reducing the level of DNA damage repair. This could explain why patients with KEAP1/NFE2L2 mutations lack DDR gene expression. Luo et al. reported a negative trend in the association between the GSEA enrichment score of DDR-related pathways and TMB (24), which also validates a higher TMB in KEAP1/NFE2L2 with mutations than in wild-type due to the lack of DDR gene expression. In addition, previous studies have indicated that patients who smoke have higher TMB levels (25). Therefore, a higher proportion of smokers in patients with mutant KEAP1/NFE2L2 may lead to an increase in TMB level.

In combination with the results of previous studies and the present study, the KEAP1/NFE2L2 mutation, as a poor prognostic factor for NSCLC, is associated with poor prognosis with targeted therapy (26), chemotherapy (2–5), or immunotherapy (6–11). But until now little success has been achieved in developing safe and effective KEAP1/NFE2L2 inhibitors for cancer therapy. Therefore, it is of great clinical significance to find a treatment method with relatively good prognosis for patients with KEAP1/NFE2L2 mutations among existing treatment methods. In this study, we compared the prognostic differences between chemotherapy and immunotherapy in patients with KEAP1/NFE2L2 mutation using datas from the OAK/POPLAR cohorts. In this study, compared with docetaxel, atezolizumab did not prolong mOS in patients with KEAP1/NFE2L2 mutations. This suggested that immunotherapy did not significantly improve prognosis compared to chemotherapy in patients with KEAP1/NFE2L2 mutations.

Our study has several limitations. First, to overcome the inadequacy of data from the OAK and POPLAR cohort for mechanism analysis, we introduced TCGA NSCLC transcriptome and in-house Chinese NSCLC datas into our study to analyze the impact of gene mutations. However, differences between these three databases may lead to bias and heterogeneity, which limits the clinical interpretation and clinical application of our study results. Second, only survival data of immunotherapy and chemotherapy were used in this study, it was not possible to analyze the prognostic difference between immunotherapy and other treatments other than chemotherapy in KEAP1/NFE2L2 mutation patients. It has implications for the search for relatively superior therapies in patients with KEAP1/NFE2L2 mutations.

In conclusion, the KEAP1/NFE2L2 mutation, as a poor prognostic factor for NSCLC, is associated with poor prognosis with immunotherapy or chemotherapy. Further research should be undertaken to investigate new therapeutic targets for patients with mutated KEAP1/NFE2L2.

## Data Availability Statement

TCGA NSCLC cohort comes from public data website: https://portal.gdc.cancer.gov/projects. The original data of in-house Chinese NSCLC cohort was provided by 3D Medicines Inc., Shanghai, China. If you need to use this part of the data, please contact the corresponding author, zhouhaiyu@gdph.org.cn.

## Author Contributions

Conception and design: HoZ, YY, and DX. Acquisition of data: LY, BX, LH, SW, and FL. Analysis and interpretation of data: WX, XL, MH, DZ, and GQ. Writing, review, and/or revision of the manuscript: HoZ, YZ, HL, and SZ. Study supervision: HaZ, YY, and LC. All authors contributed to the article and approved the submitted version.

## Funding

This study was funded by the Natural Science Foundation of Guangdong (Grant Number 2018A0303130113), Science and Technology Program of Guangzhou (Grant Number 201903010028) and Guangdong Provincial People’s Hospital Intermural Program (Grant Number KJ012019447).

## Conflict of Interest

YZ, XL, WX, and MH are employed by the company 3D Medicines Inc.

The remaining authors declare that the research was conducted in the absence of any commercial or financial relationships that could be construed as a potential conflict of interest.

## Publisher’s Note

All claims expressed in this article are solely those of the authors and do not necessarily represent those of their affiliated organizations, or those of the publisher, the editors and the reviewers. Any product that may be evaluated in this article, or claim that may be made by its manufacturer, is not guaranteed or endorsed by the publisher.

## Abbreviations

NSCLC, non-small cell lung cancer; TCGA, The Cancer Genome Atlas; GSEA, gene set enrichment analysis; KEAP1, Kelch-like ECH-associated protein 1; NFE2L2, nuclear factor erythroid-2-related factor 2; OS, overall survival; NK, natural killer; NES, normalized enrichment score; HR, hazard ratio; TMB, tumor mutation burden; DCR, disease control rate; DDR, DNA damage response; ROS, reactive oxygen species.
