# Supplementary material for: KEAP1/NFE2L2 Mutations of Liquid Biopsy as Prognostic Biomarkers in Patients With Advanced Non-Small Cell Lung Cancer: Results From Two Multicenter, Randomized Clinical Trials
Source: Front Oncol. 2021 Jul 26;11:659200. doi: 10.3389/fonc.2021.659200 (PMC8350725; doi:10.3389/fonc.2021.659200)
Supplement: Supplementary file 1 [file Presentation_1.pptx]

## Slide 1
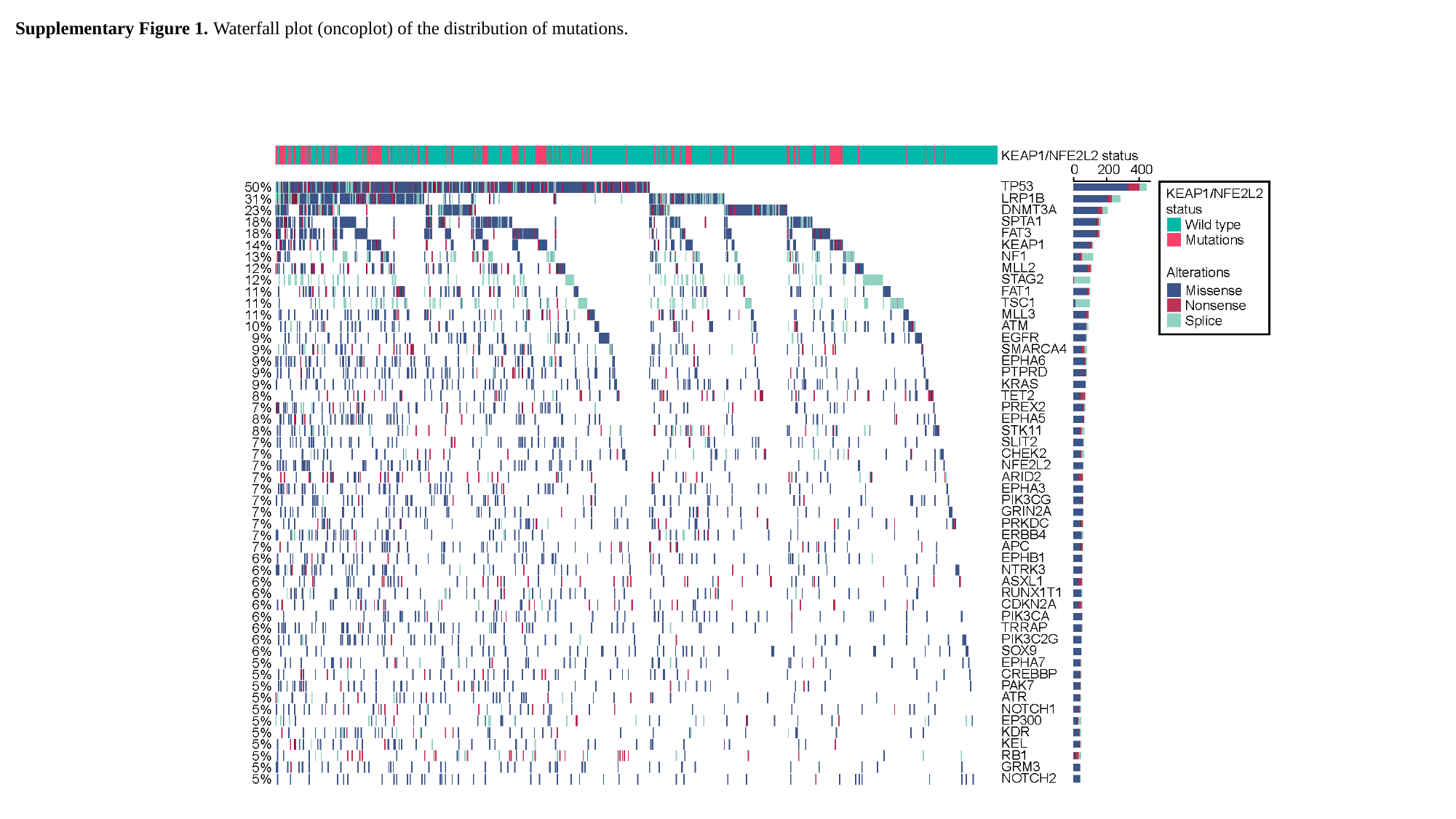

Supplementary Figure 1. Waterfall plot (oncoplot) of the distribution of mutations.

## Slide 2
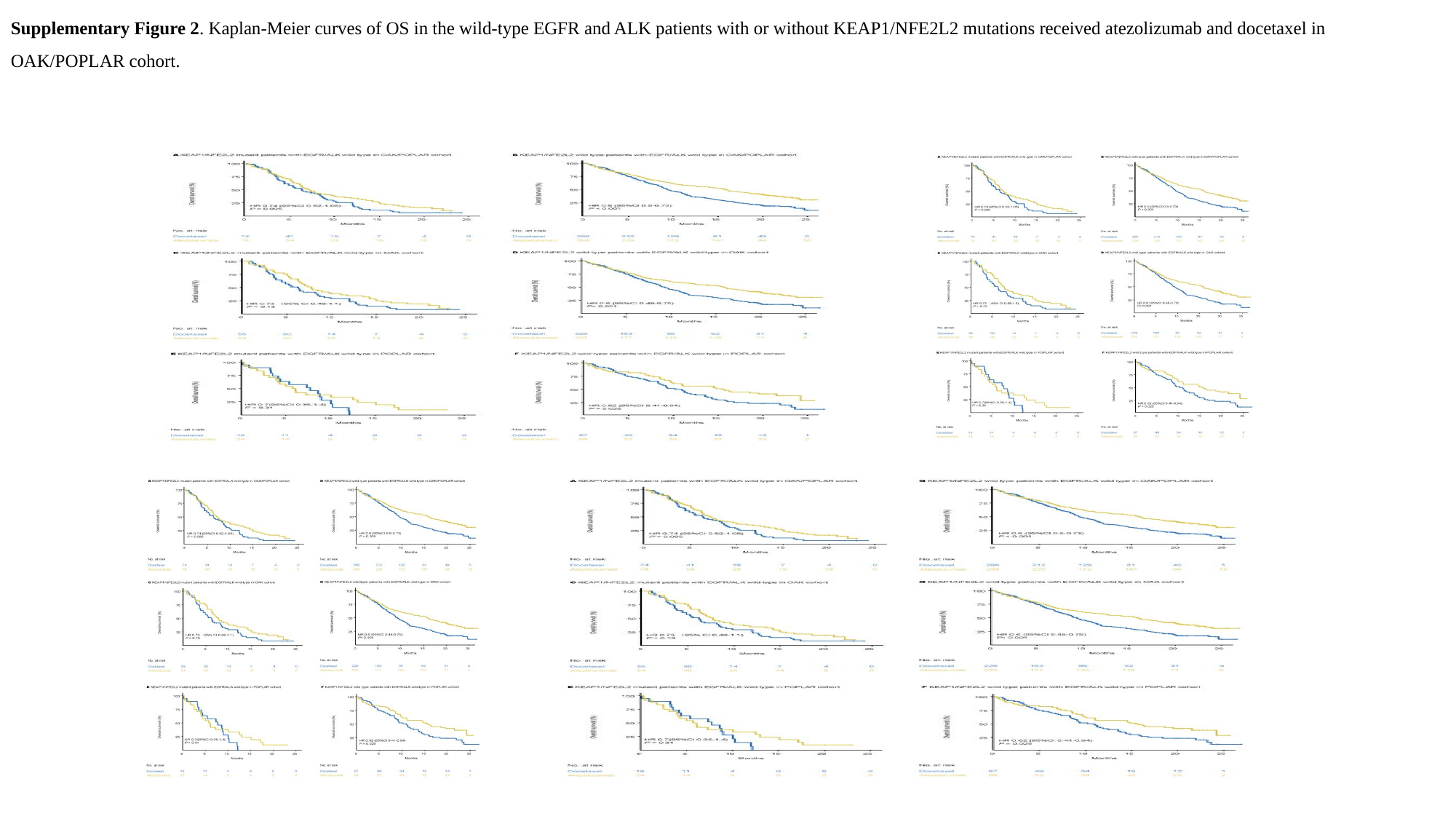

Supplementary Figure 2. Kaplan-Meier curves of OS in the wild-type EGFR and ALK patients with or without KEAP1/NFE2L2 mutations received atezolizumab and docetaxel in OAK/POPLAR cohort.

## Slide 3
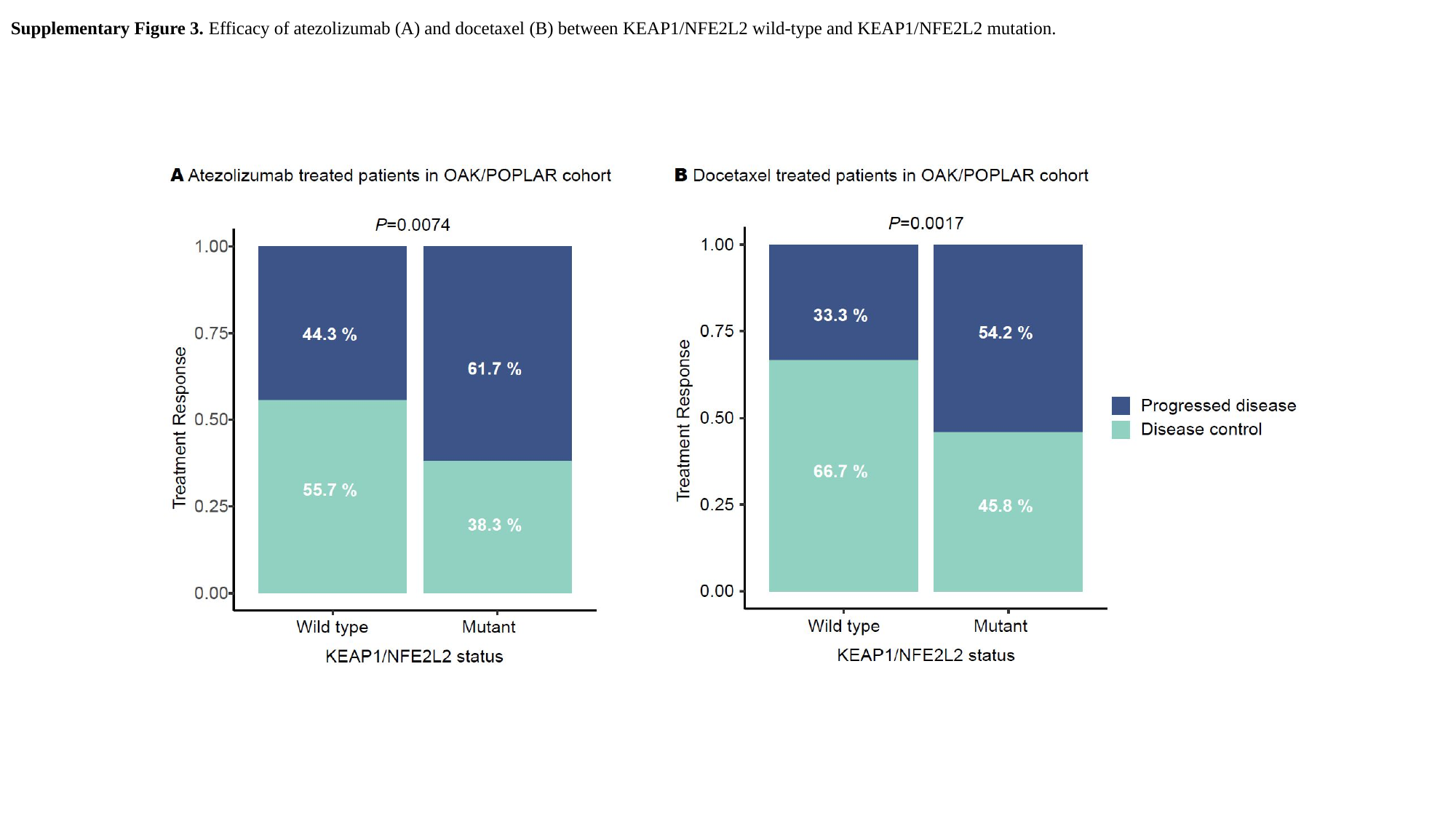

Supplementary Figure 3. Efficacy of atezolizumab (A) and docetaxel (B) between KEAP1/NFE2L2 wild-type and KEAP1/NFE2L2 mutation.

## Slide 4
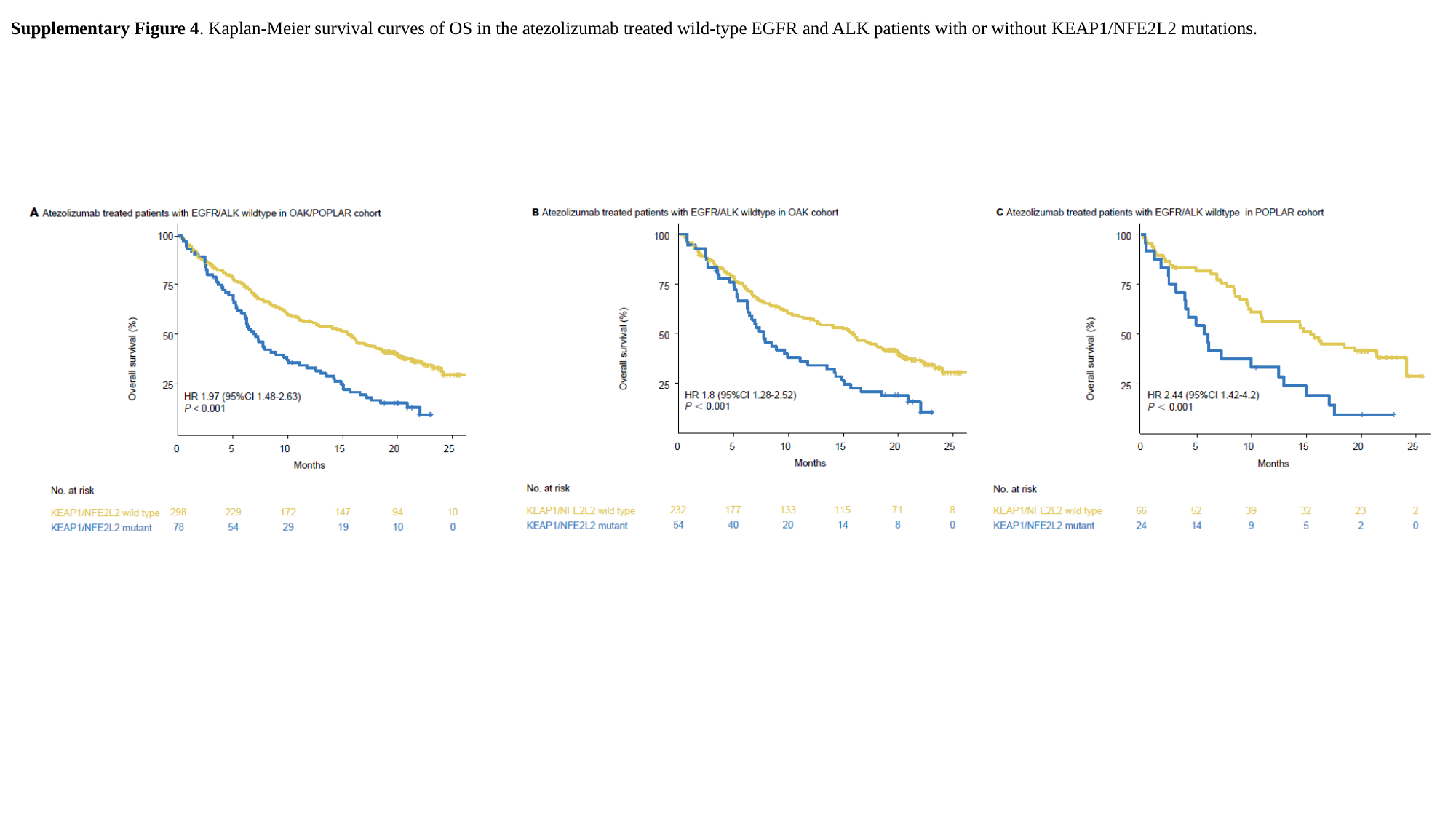

Supplementary Figure 4. Kaplan-Meier survival curves of OS in the atezolizumab treated wild-type EGFR and ALK patients with or without KEAP1/NFE2L2 mutations.

## Slide 5
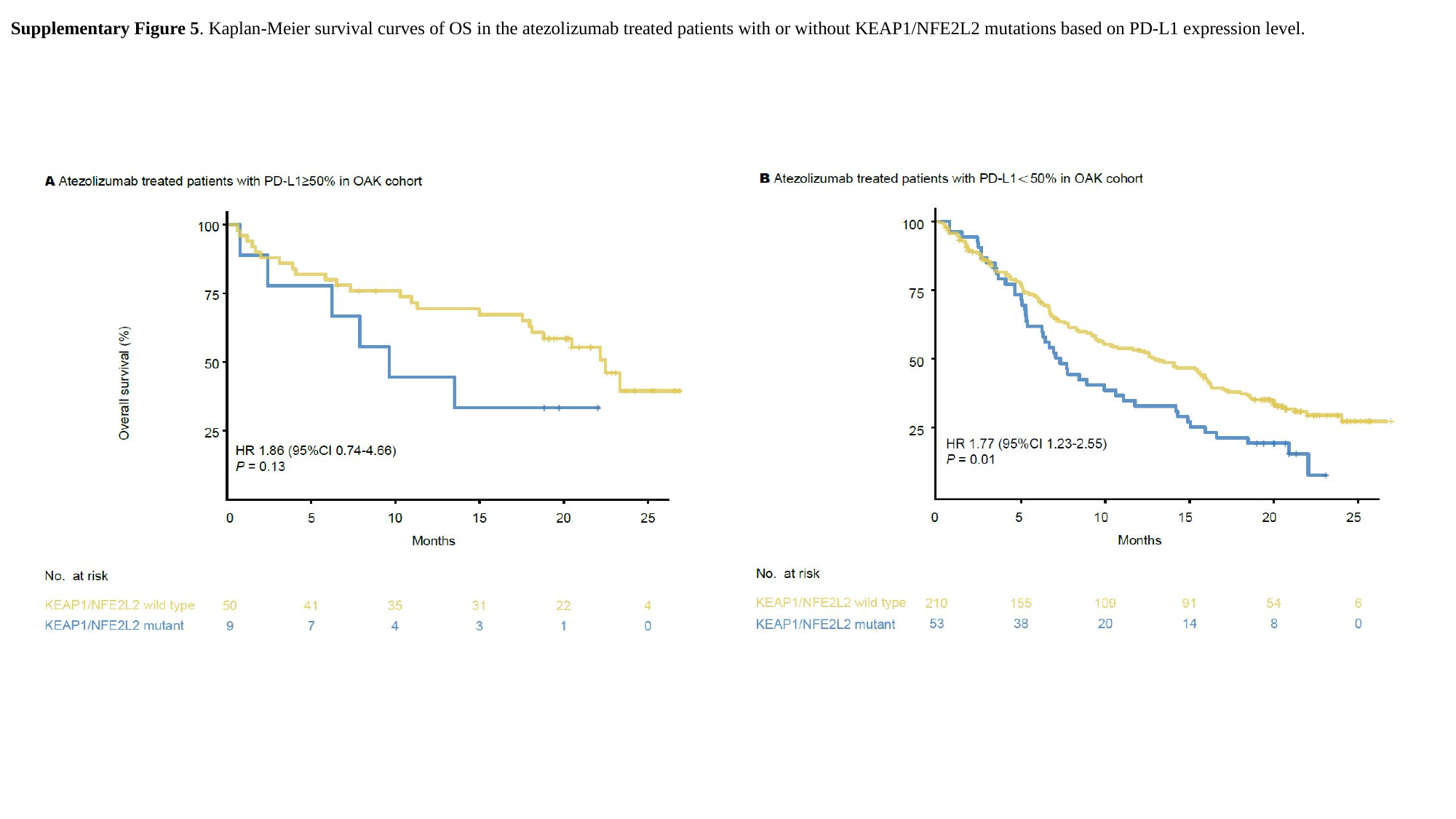

Supplementary Figure 5. Kaplan-Meier survival curves of OS in the atezolizumab treated patients with or without KEAP1/NFE2L2 mutations based on PD-L1 expression level.

## Slide 6
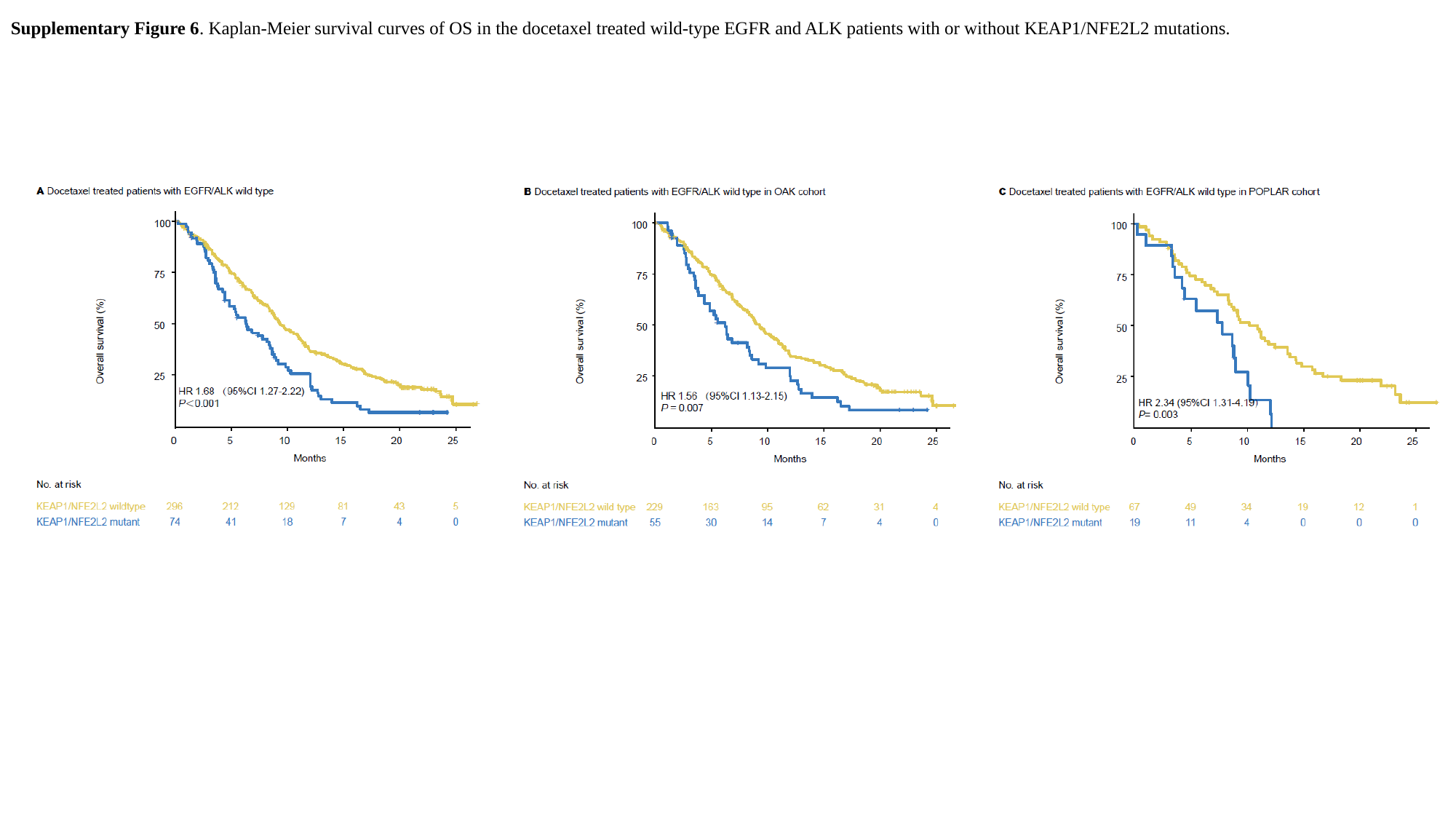

Supplementary Figure 6. Kaplan-Meier survival curves of OS in the docetaxel treated wild-type EGFR and ALK patients with or without KEAP1/NFE2L2 mutations.

## Slide 7
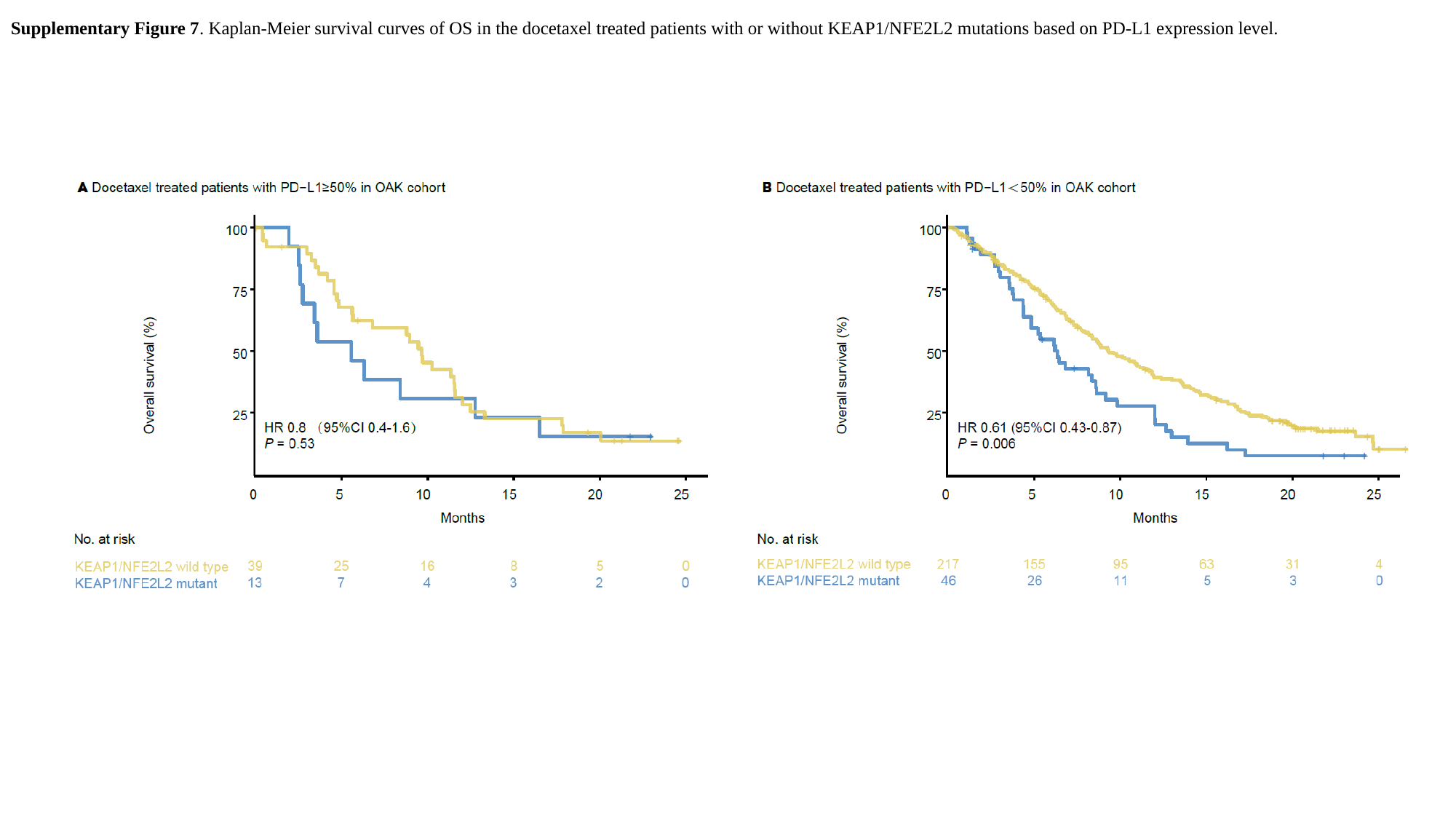

Supplementary Figure 7. Kaplan-Meier survival curves of OS in the docetaxel treated patients with or without KEAP1/NFE2L2 mutations based on PD-L1 expression level.

## Slide 8
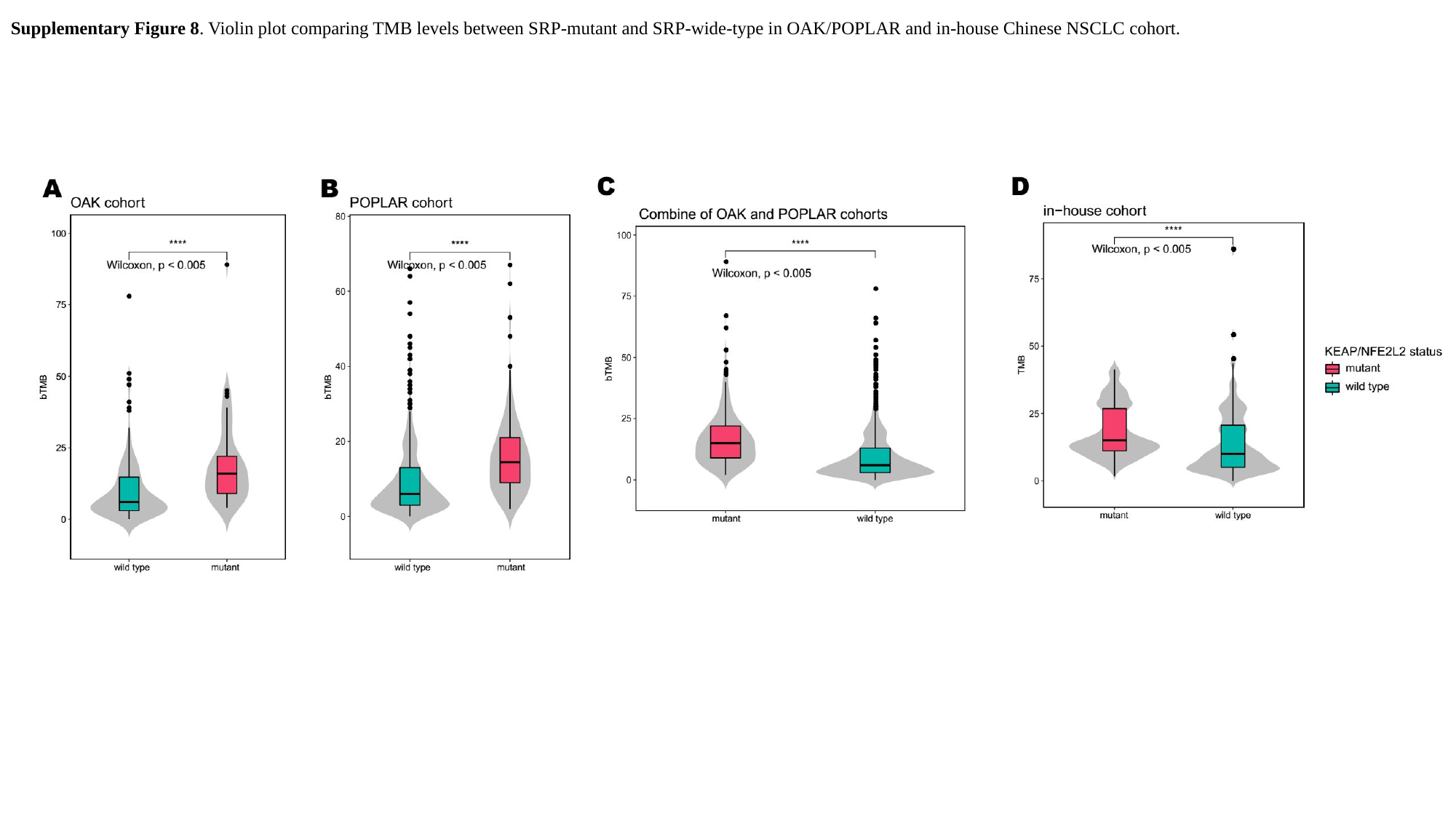

Supplementary Figure 8. Violin plot comparing TMB levels between SRP-mutant and SRP-wide-type in OAK/POPLAR and in-house Chinese NSCLC cohort.

## Slide 9
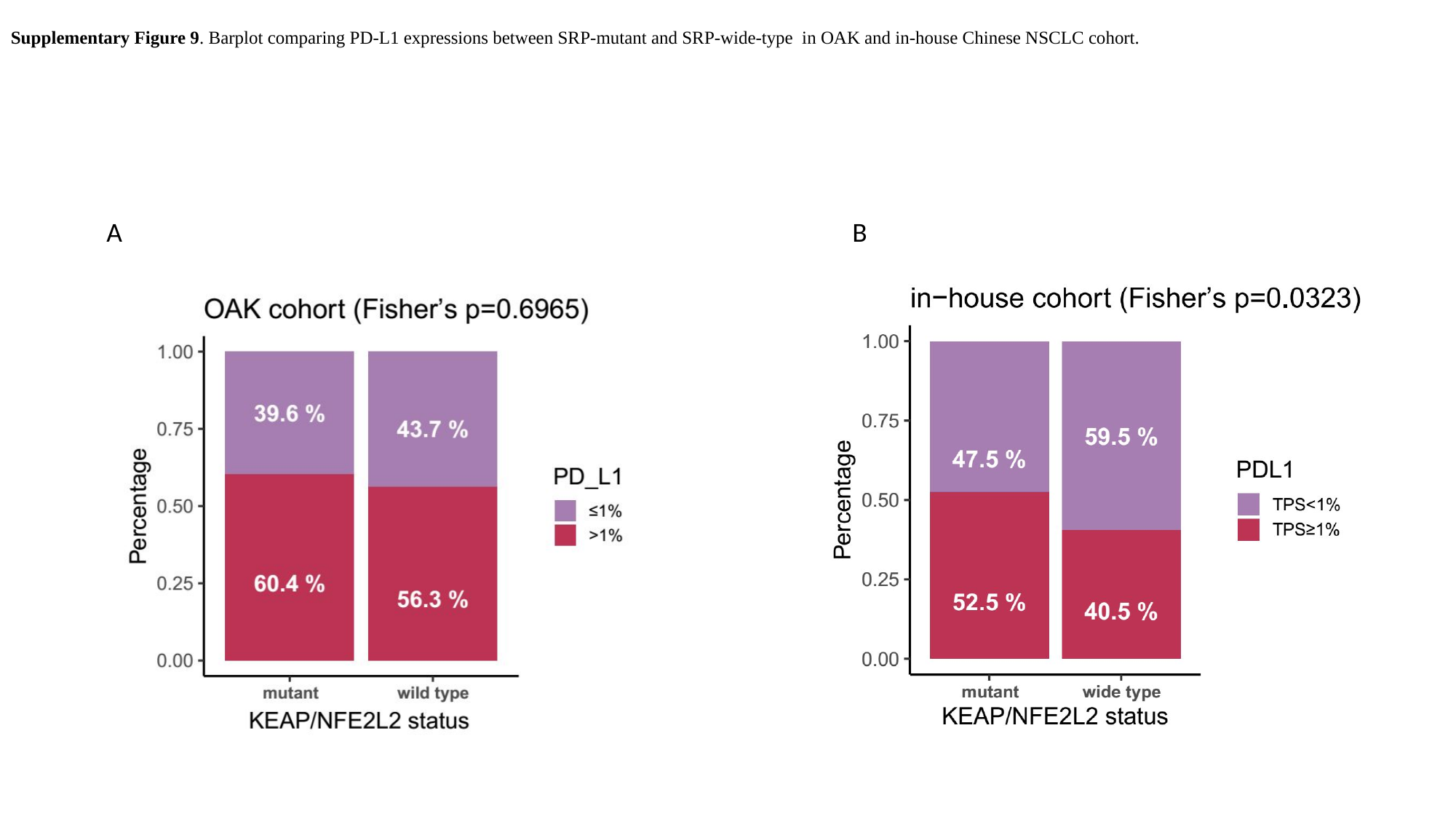

Supplementary Figure 9. Barplot comparing PD-L1 expressions between SRP-mutant and SRP-wide-type in OAK and in-house Chinese NSCLC cohort.
A
B
