# Supplementary material for: KEAP1/NFE2L2 Mutations of Liquid Biopsy as Prognostic Biomarkers in Patients With Advanced Non-Small Cell Lung Cancer: Results From Two Multicenter, Randomized Clinical Trials
Source: Front Oncol. 2021 Jul 26;11:659200. doi: 10.3389/fonc.2021.659200 (PMC8350725; doi:10.3389/fonc.2021.659200)
Supplement: Supplementary file 2 [file Table_1.docx]

**Supplementary Table 1.** Gene set enrichment analysis (GSEA) of DNA damage response (DDR) related gene sets and immunologic gene sets.

| **Name of gene signature** | **Function** | **No. of genes** | **NES** | **P value** |
| --- | --- | --- | --- | --- |
| REACTOME_BASE_EXCISION_REPAIR | Base excision repair | 87 | -1.73 | 0.0032 |
| REACTOME_DNA_REPAIR | DNA repair | 303 | -1.72 | 0.0042 |
| REACTOME_FANCONI_ANEMIA_PATHWAY | Fanconi anemia pathway | 30 | -1.6 | 0.0209 |
| REACTOME_G2_M_DNA_DAMAGE_CHECKPOINT | Checkpoint factors | 95 | -1.92 | 0.0032 |
| REACTOME_HDR_THROUGH_HOMOLOGOUS_RECOMBINATION_HRR | Homologous recombination repair | 67 | -1.69 | 0.0062 |
| REACTOME_NUCLEOTIDE_EXCISION_REPAIR | Nucleotide excision repair | 109 | -1.57 | 0.0067 |
| GSE14415_INDUCED_VS_NATURAL_TREG_DN | Down-regulated in activated Treg | 188 | -1.83 | 0.0042 |
| GSE28726_NAIVE_VS_ACTIVATED_CD4_TCELL_DN | Up-regulated in activated CD4 T cell | 199 | -1.97 | 0.0043 |
| GSE28726_NAIVE_VS_ACTIVATED_CD4_TCELL_UP | Down-regulated in activated CD4 T cell | 200 | 1.821 | 0.0013 |
| GSE28726_NAIVE_VS_ACTIVATED_NKTCELL_UP | Down-regulated in activated NKT cell | 199 | 1.42 | 0.0052 |
